# Alcohol and Alcoholic Preparations in Surgery

**Published:** 1860-03

**Authors:** 


*Alcohol and Alcoholic Preparations in Surgery.*

We are indebted to Prof. T. Deville, of the Lind University of this City, for an early copy of the above monograph, which has been transmitted to him by the Author, on the use of Alcohol and Alcoholic Preparations in Surgery.

After having examined the subject by the threefold light of theory, experiment, and history; the following are the results arrived at:

"Alcohol coagulates albumen in whatever liquid it may be, and consequently in blood, the synovia of synovial membranes, of articulations, the serosity which bathes the meshes of cellular tissue, and that which moistens the surfaces of serous visceral cavities.

Alcohol applied to living tissues on the surface of a wound provokes no sort of accident. We are convinced of this from repeated experiments.

It coagulates albumen on the surface of wounds, forming it into a greyish-white-pellicule. It stops the hemorrhage of the small vessels. It hastens the secretion of plastic lymph on the surface of the wounds. By separating the lips of the wound soon after the application of the alcohol, we can watch the lymph in the very act of gluing them together; that is to say, the same thing is taking place there which takes place on the surface of serous membranes.

From these facts and these principles it results that alcohol exercises a great influence on union by the first intention, that it prevents diffuse erysipelatous inflammation, purulent infusion of synovial membranes, and purulent infection.

Let us explain ourselves:

Alcohol favors union by the first intention, by stopping hemorrhage of the smaller vessels (since the blood is a great obstacle to a perfect coaptation of the parts) by immediately producing a coagulum on the surface of wounds, and by accelerating the plastic secretion.

It prevents diffuse suppuration by coagulating the albumen of the cellular tissue, which immediately becomes thick, and is rendered impermeable to the unhealthy liquids which bathe their surfaces.

Alcohol prevents the purulent effusions of synovial membranes by the immediate coagulation of the synovia, by causing the opposite surfaces to adhere, by rendering them impermeable to the liquids which moisten the surfaces of fresh wounds, and by encouraging an adhesive inflammation of the synovial membranes near the wound.

Alcohol prevents purulent infection, by coagulating the blood in the open and even gaping veins, on the surfaces of wounds, thus instantly plugging them and favoring an adhesive inflammation." * * * * * *

We must, therefore, in dressing recent wounds abandon the use of fatty bodies and poultices, and return to alcoholic preparations; in a word we must return to the practice of the ancients.

